# A Glandular Odontogenic Cyst Mimicking a Dentigerous Cyst: A Diagnostic Pitfall

**DOI:** 10.7759/cureus.111329

**Published:** 2026-06-22

**Authors:** Aryan Wadehra, Sahana Srinath, Vaishnavi N Setloor, Akhila S, Anjana K

**Affiliations:** 1 Oral Pathology and Microbiology, Government Dental College and Research Institute, Bengaluru, IND

**Keywords:** dentigerous relationship, differential histopathological diagnosis, glandular odontogenic cyst, odontogenic cyst, pericoronal radiolucency

## Abstract

Glandular odontogenic cysts (GOCs) are rare developmental odontogenic cysts that may pose significant diagnostic challenges due to their overlapping clinical and radiographic features with other odontogenic lesions. We report a case of a 29-year-old woman who presented with pain and restricted mouth opening associated with an impacted right mandibular third molar. Cone-beam computed tomography revealed a well-defined pericoronal radiolucency attached to the crown of the impacted tooth and extending to the distal root of the adjacent second molar. Based on the characteristic pericoronal location and apparent attachment at the cemento-enamel junction, a provisional diagnosis of a dentigerous cyst was made. Surgical enucleation of the lesion and extraction of the associated tooth were performed. Gross examination demonstrated continuity between the cystic lining and the cervical region of the tooth. Histopathological evaluation revealed a non-keratinised odontogenic epithelial lining of variable thickness with epithelial plaque-like thickenings, intraepithelial microcysts, superficial eosinophilic cuboidal (hobnail) cells exhibiting apocrine snouting, clear cells, and ciliated cells, consistent with a definitive histopathological diagnosis of a GOC. This case is notable because the dentigerous relationship was consistently demonstrated radiographically, grossly, and microscopically, yet the lesion exhibited definitive histopathological features of a GOC. The findings highlight the limitations of relying solely on clinicoradiographic features, including attachment at the cemento-enamel junction, for diagnosing pericoronal lesions. Careful histopathological assessment remains essential for distinguishing GOCs from dentigerous cysts and other lesions with overlapping features, ensuring accurate diagnosis and appropriate management.

## Introduction

Glandular odontogenic cysts (GOCs) are uncommon odontogenic cysts. They are defined as developmental odontogenic cysts with an epithelial lining that shows features of glandular differentiation [1]. Since their initial description as sialo-odontogenic cysts and subsequent recognition as a distinct entity, GOCs have been reported infrequently. They are a rare entity, comprising less than 0.5% of odontogenic cysts [2]. They show a greater predilection for the anterior mandible and for middle-aged individuals [3]. Despite well-defined microscopic features, diagnosis remains challenging due to their variable clinical and radiographic presentations [2]. 

Radiographically, GOCs typically present as well-defined, unilocular or multilocular radiolucencies. They are often associated with cortical expansion and can occasionally be found with impacted teeth. These features frequently overlap with those of several odontogenic cysts and tumours, especially dentigerous cysts, odontogenic keratocysts, and ameloblastomas [4]. Moreover, the concept of a “dentigerous relationship,” in which GOC is associated with the crown of an unerupted tooth, has been increasingly recognised, thereby further complicating clinical diagnosis [5].

Dentigerous cysts are classically defined by their attachment at the cemento-enamel junction of an unerupted tooth. This is considered a primary diagnostic criterion in clinicoradiographic assessment [6]. As a result, pericoronal radiolucencies attached to the cemento-enamel junction are frequently interpreted with high confidence as dentigerous cysts. However, reliance on anatomical relationships and radiographic appearance alone can be misleading. This is especially true for lesions that exhibit overlapping biological and histopathological characteristics.

In this context, we report a case of a GOC arising in the posterior mandibular third molar region of a 29-year-old patient. The lesion was attached to the cemento-enamel junction and exhibited radiographic features suggestive of a dentigerous cyst but showed definitive histopathological features of a GOC. Given its atypical age and site of presentation, the case highlights a potential diagnostic pitfall and emphasises the need for careful histopathological evaluation, even when lesions display typical clinicoradiographic features.

## Case presentation

A 29-year-old female patient was referred to our institute from a private dental clinic with a chief complaint of pain in the lower right posterior region. The pain was associated with restricted mouth opening (trismus) and an unerupted mandibular third molar (tooth 48). The trismus was considered to be related to the impacted third molar rather than the cystic lesion itself. She had no history of associated swelling, discharge of pus or blood, and no significant extraoral or intraoral swelling was noted during clinical examination.

Cone-beam computed tomography was performed to evaluate the impacted third molars. The imaging showed a horizontally impacted mandibular right third molar (48) and a mesioangularly impacted mandibular left third molar (38). A well-defined ovoid hypodense lesion was noted surrounding the crown of tooth 48, suggestive of a cystic lesion. The lesion measured approximately 13.8 × 15.4 × 9.7 mm and extended to the distal root of tooth 47, with associated bone loss. The mesial aspect of the crown of 48 was in close contact with the distal surface of 47. No root resorption or caries was observed in relation to 47 (Figure 1).

**Figure 1:**
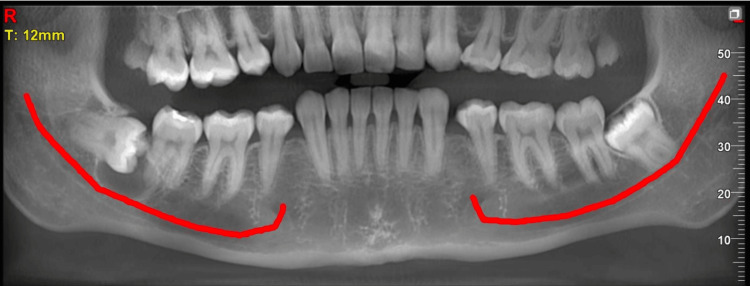
Reconstructed panoramic view from cone-beam computed tomography (CBCT), demonstrating a well-defined pericoronal radiolucency associated with an impacted mandibular right third molar (48). The red line indicates the radiographically identified course of the mandibular canal.

Tooth 48 exhibited complete root formation with two roots and two canals, with the root apices in close proximity to the mandibular canal. The surrounding buccal and lingual cortical plates did not show significant thinning (Figure 2).

**Figure 2:**
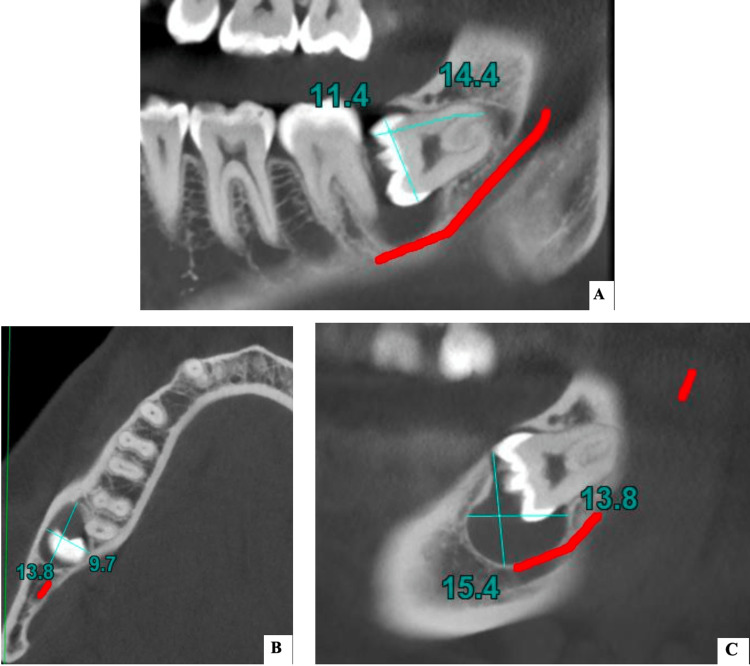
Cone-beam computed tomography (CBCT) views of the mandibular right third molar (48): (A) sagittal view, (B) axial view, and (C) cross-sectional view. Linear measurements indicate the approximate dimensions of the lesion. The red line indicates the radiographically identified course of the mandibular canal.

Based on these clinicoradiographic findings, a provisional diagnosis of a dentigerous cyst associated with impacted tooth 48 was made. Surgical enucleation of the cystic lesion was performed, along with the extraction of tooth 48. The tooth was sectioned and removed. The cystic lining, which was attached to the tooth, was retrieved in toto. The excised specimen, which comprised the sectioned tooth and attached cystic lining, was submitted for histopathological examination.

On gross examination, a cystic lining attached to the cervical region of tooth 48 was obtained. The soft tissue specimen measured approximately 1.3 × 1.1 × 0.9 cm. The entire specimen, the tooth with attached cystic lining, was processed for microscopic evaluation (Figures 3, 4).

**Figure 3:**
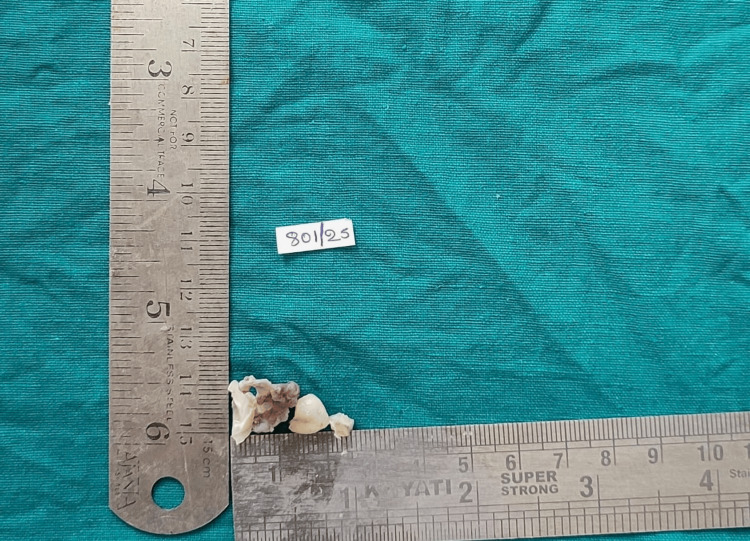
Gross specimen showing a sectioned mandibular right third molar (48) with cystic lining attached to the cervical portion of the tooth, along with additional sectioned fragments of the tooth. The linear scale is included for size reference.

**Figure 4:**
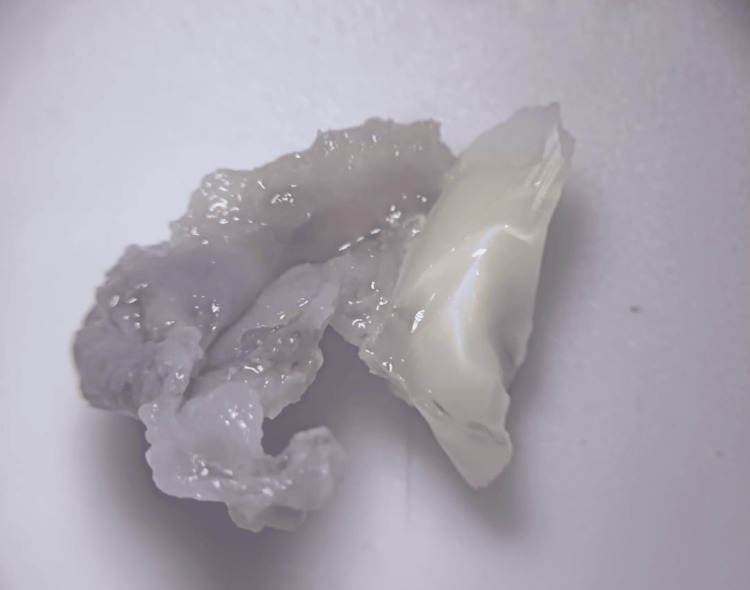
Stereomicroscopic image showing cystic lining attached to a section of the mandibular right third molar (48), demonstrating continuity between the cyst wall and the tooth structure.

Microscopic examination of decalcified hematoxylin and eosin-stained sections revealed a cystic lesion lined by odontogenic epithelium supported by an underlying connective tissue capsule. The cystic lining was noted to be attached to the cemento-enamel junction of the associated tooth at one end (Figures 5, 6).

**Figure 5:**
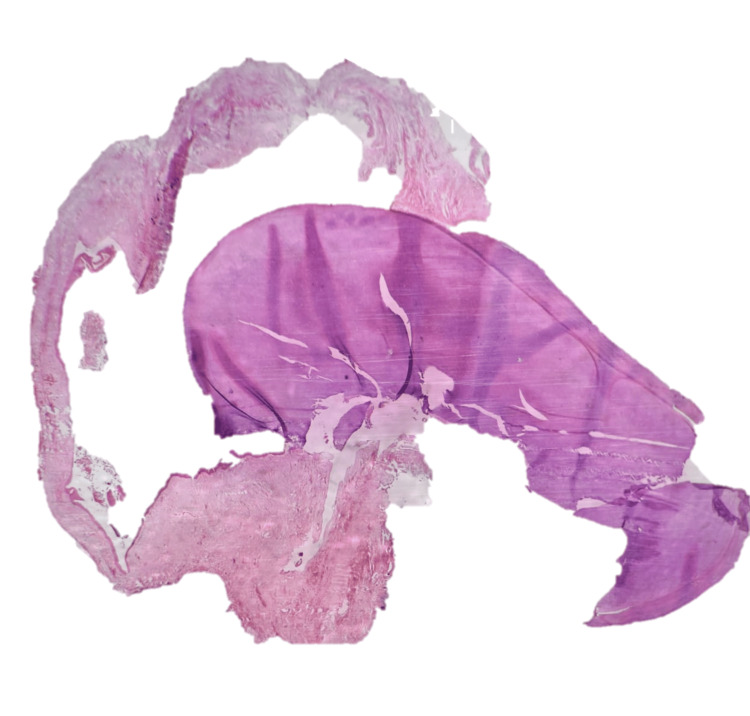
Low-power photomicrograph showing the H&E-stained tooth section surrounded by cystic lining in continuity, demonstrating the relationship between the cyst wall and tooth structure.

**Figure 6:**
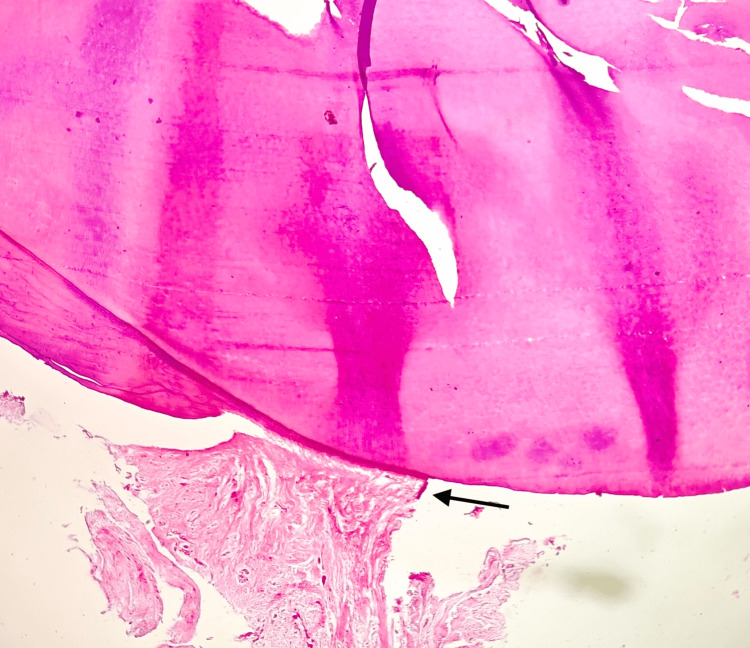
Photomicrograph showing attachment of the cystic lining to the tooth (black arrow), with an underlying fibrous connective tissue capsule (H&E stain, 100x).

The epithelial lining was non-keratinised stratified squamous. It exhibited variable thickness (Figure 7), ranging from flattened to cuboidal cells, with focal areas showing epithelial plaque-like thickenings. Numerous intraepithelial microcystic spaces were evident (Figure 8). The superficial layer demonstrated eosinophilic cuboidal cells with characteristic hobnail morphology. These cells showed apocrine snouting. Occasional ciliated cells were also observed. Additionally, scattered clear cells were present (Figures 9-11).

**Figure 7:**
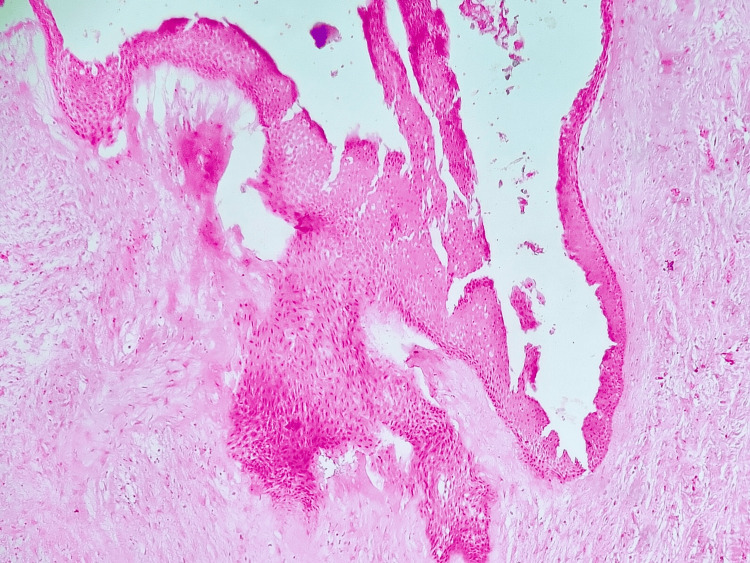
Photomicrograph showing non-keratinized stratified squamous epithelial lining of variable thickness with an underlying fibrous connective tissue capsule (H&E stain, 100x).

**Figure 8:**
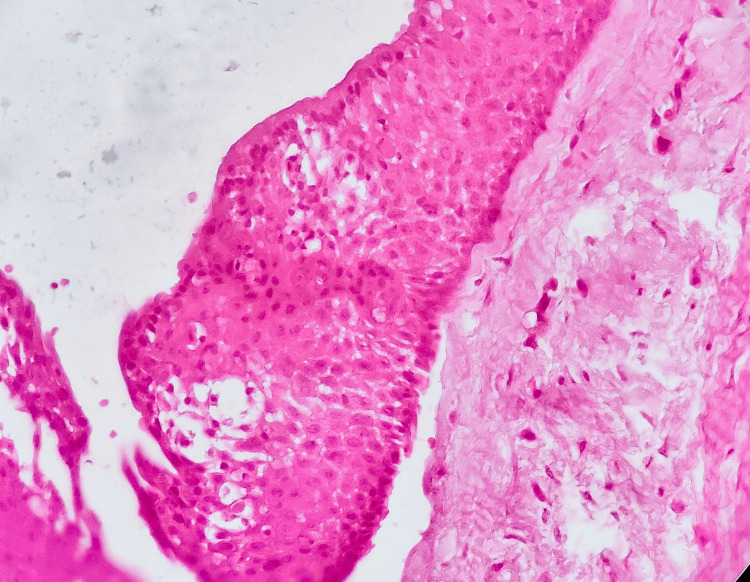
Photomicrograph showing odontogenic cystic lining with epithelial plaque-like thickening, intraepithelial microcystic spaces, and superficial eosinophilic cuboidal (hobnail) cells (H&E stain, 400x).

**Figure 9:**
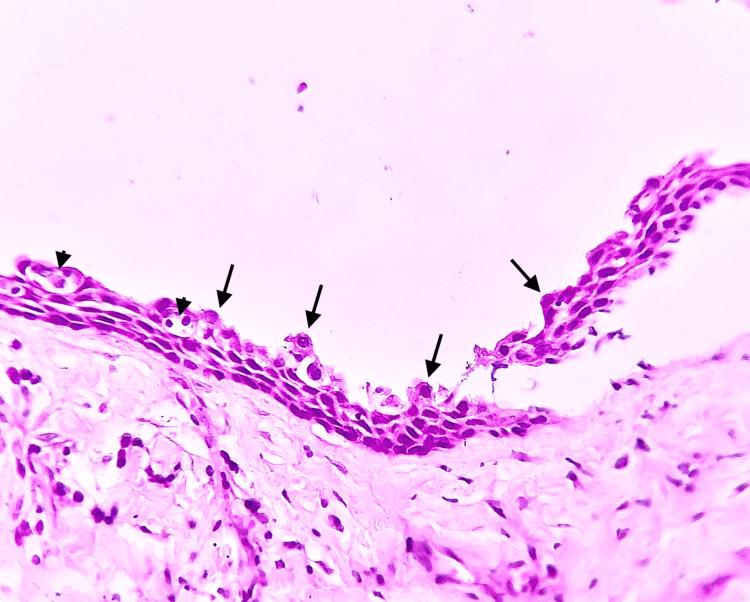
Photomicrograph showing non-keratinized cystic epithelial lining with superficial eosinophilic cuboidal hobnail cells (black arrows) and clear cells (black arrowheads) (H&E stain, 400x).

**Figure 10:**
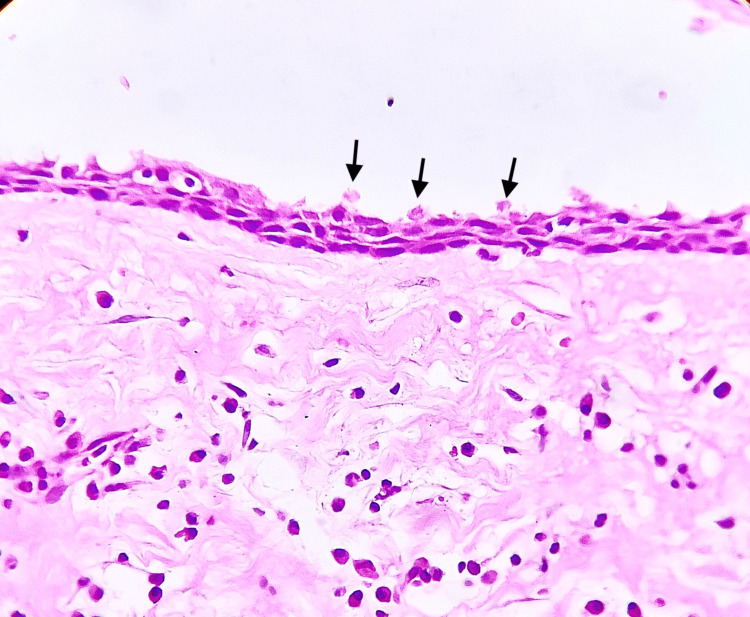
Photomicrograph showing non-keratinized cystic epithelial lining with superficial eosinophilic cuboidal hobnail cells with apocrine snouting (black arrows) and clear cells (H&E stain, 400x).

**Figure 11:**
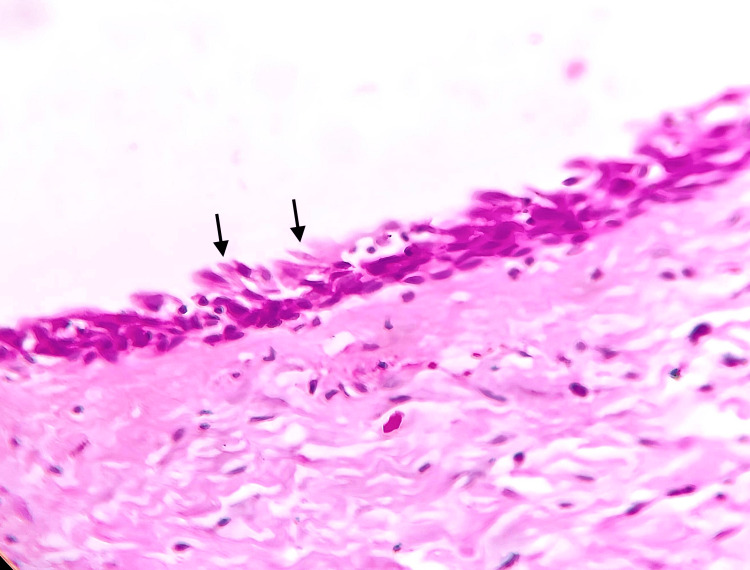
Photomicrograph showing cystic epithelial lining with the presence of ciliated cells (black arrows) and clear cells within the epithelium (H&E stain, 400x).

The underlying connective tissue capsule was composed of dense collagen fiber bundles. These were interspersed with fibroblasts and fibrocytes. Numerous endothelial-lined blood vessels were present, some of which were engorged with red blood cells. A moderate chronic inflammatory infiltrate was also noted, predominantly comprising lymphocytes and plasma cells.

The present case demonstrated seven of the 10 microscopic parameters described by Fowler et al., including hobnail cells, intraepithelial microcysts, apocrine snouting, clear cells, variable epithelial thickness, epithelial plaque-like thickenings, and ciliated cells [7], thus fulfilling the diagnostic threshold strongly predictive of a GOC, and a final diagnosis of a GOC was made.

Follow-up three months after the procedure revealed no early recurrence, and in view of the diagnosis of the GOC, the patient has been placed on a six-month follow-up schedule. 

## Discussion

The present case demonstrated a pericoronal radiolucency associated with an impacted mandibular third molar, showing attachment at the cemento-enamel junction, and was provisionally diagnosed as a dentigerous cyst. This presentation corresponds to what Fowler et al. described as a “dentigerous relationship,” wherein a GOC is associated with the crown of an unerupted tooth and may mimic a dentigerous cyst clinically and radiographically. In their series of 46 cases, Fowler et al. reported that eight cases exhibited such a dentigerous relationship, highlighting the potential for diagnostic overlap between these two entities [7].

Several case reports in the literature have documented a GOC presenting in association with unerupted teeth and mimicking a dentigerous cyst both clinically and radiographically. These lesions are most commonly described as well-defined unilocular radiolucencies surrounding the crown of an impacted tooth, often leading to an initial diagnosis of a dentigerous cyst prior to histopathological evaluation [8]. The radiographic appearance of GOCs is considered nonspecific and not pathognomonic, with features overlapping with a variety of odontogenic cysts and tumors, thereby making preoperative diagnosis difficult [9].

The concept of a GOC occurring in a dentigerous relationship (GOC-DR) has been increasingly recognized in the literature, although only a limited number of such cases have been reported. These cases demonstrate a wide clinical spectrum, with lesions occurring predominantly in the mandible and frequently involving impacted third molars [5]. Therefore, histopathological examination using established criteria remains essential for definitive diagnosis, as the overlapping clinical and radiographic features do not allow reliable differentiation between a GOC and a dentigerous cyst.

Kaplan et al. initially proposed major and minor microscopic criteria for the diagnosis of a GOC, suggesting that all major features must be present for a definitive diagnosis, with minor criteria serving a supportive role [10]. However, this approach is limited by the inherent histological variability of the GOC, as not all characteristic features are consistently identified in every case. In particular, features such as mucous cells, although characteristic, may be absent in some cases, thereby limiting the applicability of rigid diagnostic criteria.

To overcome these limitations, Fowler et al. proposed a parameter-based diagnostic approach, emphasizing the cumulative presence of multiple microscopic features rather than mandatory inclusion of specific criteria. They suggested that the presence of a higher number of characteristic features, particularly seven or more parameters, is strongly predictive of a GOC, whereas five or fewer features favor an alternative diagnosis. They highlighted that certain features, particularly intraepithelial microcysts, clear cells, and epithelial plaques, are more discriminative in distinguishing GOCs from lesions with overlapping histological features, including dentigerous cysts with metaplastic changes [7]. The histopathological parameters thus given are described in Table 1 [7].

**Table 1:** Histopathological parameters for glandular odontogenic cysts. Diagnosis of a glandular odontogenic cyst is supported by the presence of ≥7 parameters, whereas ≤5 parameters favour a non-GOC diagnosis [7]. GOC: Glandular odontogenic cyst

Histopathological Feature	Description
Surface eosinophilic cuboidal cells (hobnail cells)	Surface cells resembling reduced enamel epithelium, often showing apical snouting
Intraepithelial microcysts/duct-like spaces	Small cystic spaces within epithelium, may contain mucous or eosinophilic material
Apocrine snouting	Decapitation-like secretion from surface hobnail cells
Clear or vacuolated cells	Glycogen-rich cells in basal or parabasal layers
Variable epithelial thickness	Marked variation in epithelial lining thickness
Papillary projections (tufting)	Luminal epithelial proliferations, sometimes arising from microcysts
Mucous (goblet) cells	Present singly or in clusters, may line microcysts
Epithelial spheres/plaque-like thickenings	Whorled or plaque-like epithelial proliferations
Multiple compartments	Multicystic or multiluminal architecture
Cilia	True cilia on surface epithelial cells

In the present case, application of the criteria proposed by Fowler et al. identified seven diagnostic parameters, thereby meeting the threshold considered strongly predictive of a GOC. Notably, mucous (goblet) cells were not identified in the present case. However, Fowler et al. demonstrated that no single histopathological feature is mandatory for the diagnosis of GOCs and that the cumulative presence of multiple characteristic parameters is more diagnostically significant. The identification of seven diagnostic parameters, including several highly discriminative features such as hobnail cells, intraepithelial microcysts, clear cells, apocrine snouting, and epithelial plaques, therefore supports the diagnosis despite the absence of mucous cells [7]. This criteria-based approach is particularly important in lesions associated with unerupted teeth, where overlapping features with dentigerous cysts may lead to diagnostic uncertainty.

The principal differential diagnosis in the present case is a dentigerous cyst with mucous metaplasia, particularly given the pericoronal location and attachment to the cemento-enamel junction. Dentigerous cysts may exhibit metaplastic changes, including mucous cells and ciliated cells, which can simulate a GOC [11]. However, such changes are usually limited, and the lesion lacks the combination of multiple characteristic features required for a diagnosis of a GOC [5]. In addition, intraepithelial mucus pools in dentigerous cysts may resemble microcysts; however, these are typically lined by flattened epithelial cells and do not represent true duct-like structures. In contrast, a GOC demonstrates true microcysts lined by cuboidal or columnar cells [12]. In the present case, the presence of several discriminative features, including superficial cuboidal hobnail cells, apocrine snouting, intraepithelial microcysts, clear cells, and epithelial plaques, favors a GOC over a dentigerous cyst with metaplastic changes. A comparative summary of the distinguishing histopathological features of dentigerous cysts, GOCs, and the findings observed in the present case is provided in Table 2.

**Table 2:** Comparison of histopathological findings of the dentigerous cyst, glandular odontogenic cyst, and the present case.

Histopathological feature	Dentigerous cyst	Glandular odontogenic cyst	Present case
Variable epithelial thickness	Uncommon	Characteristic	Present
Hobnail (eosinophilic cuboidal) cells	Absent	Characteristic	Present
Apocrine snouting	Absent	Characteristic	Present
Intraepithelial microcysts/duct-like spaces	Absent	Characteristic	Present
Clear/vacuolated cells	Rare	Characteristic	Present
Mucous cells	May occur as metaplasia	Common	Absent
Ciliated cells	May occur as metaplasia	May be present	Present
Epithelial plaques	Absent	Characteristic	Present
Number of Fowler parameters	Not applicable	≥7 supports diagnosis	7 identified

Lateral periodontal cysts (LPCs) may be included in the differential diagnosis due to the presence of clear cells and focal epithelial thickenings. However, LPCs typically occur along the lateral root surface of teeth and lacks glandular features such as intraepithelial microcysts, hobnail cells, mucous cells, and ciliated cells. The presence of these features in the present case favors a diagnosis of a GOC. Botryoid odontogenic cysts, a multicystic variant of LPCs, may exhibit epithelial plaques and a multilocular architecture, leading to diagnostic overlap. However, they lack key glandular features, including microcysts, mucous cells, and apocrine snouting. In addition, epithelial plaques in GOCs are often more prominent and may form papillary projections, which are uncommon in botryoid odontogenic cysts. The constellation of features in the present case is therefore not consistent with a botryoid odontogenic cyst [12].

Low-grade central mucoepidermoid carcinoma (CMEC) is a critical differential diagnosis due to overlapping features, including mucous cells, clear cells, and a multicystic architecture. A biological continuum between GOCs and CMEC has been proposed, with reports suggesting that CMEC may arise from the epithelial lining of odontogenic cysts, including GOC [12,13]. CMEC exhibits a true neoplastic proliferation composed of mucous, epidermoid, and intermediate cells forming solid or polycystic epithelial islands infiltrating the surrounding connective tissue or bone, whereas in GOCs, similar elements are confined to the epithelial lining without stromal invasion. MECs are almost always multicystic, whereas GOCs show this appearance in only 60-70% cases. In addition, several features are characteristic of GOC and are rarely or not observed in CMEC, including ciliated cells, apocrine snouting of hobnail cells, superficial eosinophilic cuboidal (hobnail) cells, intraepithelial microcysts, and plaque-like epithelial thickenings [12]. At the molecular and immunophenotypic levels, CMEC is associated with MAML2 gene rearrangements and cytokeratin 18 expression, whereas the GOC lacks MAML2 alterations and shows cytokeratin 19 expression, further aiding in distinguishing the two entities [12,14]. In the present case, the diagnosis was established on the basis of characteristic histomorphological findings and fulfilment of the Fowler diagnostic criteria. Ancillary investigations, including immunohistochemical or molecular studies such as CK19 expression and MAML2 rearrangement analysis, were not performed. Although the observed histopathological features strongly favored GOCs over CMEC, the absence of ancillary confirmation may be recognized as a limitation of the present report.

GOCs demonstrate a recognized tendency for recurrence, reported in approximately 20-22% of cases, particularly in larger and multilocular lesions [1,12]. This behavior is attributed to its multicystic architecture and potential for incomplete removal, especially following conservative procedures such as enucleation. More extensive surgical approaches, including peripheral osteoectomy or resection, have been associated with lower recurrence rates in selected cases. Recurrences may occur after a prolonged interval, often several years following treatment, thereby necessitating long-term follow-up with periodic clinical and radiographic evaluation [15]. 

A limitation of the present report is the relatively short follow-up period of three months. Although no early recurrence was identified during this interval, the recognized potential for late recurrence in GOCs underscores the need for longer-term clinical and radiographic surveillance to fully assess treatment outcomes.

The present case offers a distinctive clinicopathological correlation in which a lesion demonstrating a classical dentigerous relationship, including cemento-enamel junction attachment, was consistently identified across radiographic, gross, and histological levels in continuity with the intact tooth-cyst complex, yet revealed definitive features of a GOC on microscopic evaluation. Such comprehensive documentation across multiple levels provides a more integrated demonstration of the discordance between anatomical presentation and histopathological diagnosis. This observation highlights the limitations of relying solely on conventional anatomical criteria in pericoronal lesions and underscores the importance of a parameter-based histopathological approach to resolve diagnostically challenging cases.

## Conclusions

GOCs occurring in a dentigerous relationship are a recognized but uncommon presentation, wherein their anatomical and radiographic resemblance to conventional dentigerous cysts poses a significant diagnostic challenge. The present case exemplifies this diagnostic pitfall, wherein a lesion demonstrating a consistent dentigerous relationship across radiographic, gross, and microscopic levels was ultimately diagnosed as GOCs on histopathological evaluation, highlighting the limitations of relying solely on clinicoradiographic findings. Importantly, attachment at the cemento-enamel junction, although classically regarded as a characteristic feature of dentigerous cysts, should not be considered pathognomonic when evaluating pericoronal radiolucent lesions.

This case emphasizes the importance of a systematic, criteria-based histopathological approach in establishing the diagnosis, particularly in lesions with overlapping features, as imaging and clinical findings alone may not reveal the constellation of glandular features that define GOCs. Careful evaluation and awareness of such similarities are essential to ensure accurate diagnosis and appropriate management, as GOCs and dentigerous cysts differ in their clinical behavior and long-term prognosis, with implications for both the extent of surgical treatment and the duration of postoperative follow-up. All enucleated pericoronal cystic specimens should therefore be submitted for histopathological examination, regardless of how convincing the preoperative diagnosis may appear, to enable correct diagnosis and institution of an appropriate follow-up protocol.
